# Effects of Meshed p-type Contact Structure on the Light Extraction Effect for Deep Ultraviolet Flip-Chip Light-Emitting Diodes

**DOI:** 10.1186/s11671-019-2984-0

**Published:** 2019-05-02

**Authors:** Yuxin Zheng, Yonghui Zhang, Ji Zhang, Ce Sun, Chunshuang Chu, Kangkai Tian, Zi-Hui Zhang, Wengang Bi

**Affiliations:** Key Laboratory of Electronic Materials and Devices of Tianjin, School of Electronics and Information Engineering, Hebei University of Technology, 5340 Xiping Road, Beichen District, Tianjin, 300401 People’s Republic of China

**Keywords:** Ultraviolet light-emitting diode, Light extraction efficiency, AlGaN, Finite-difference time-domain method, p-type contact

## Abstract

In this work, flip-chip AlGaN-based deep ultraviolet light-emitting diodes (DUV LEDs) with various meshed contact structures are systematically investigated via three-dimensional finite-difference time-domain (3D FDTD) method. It is observed that both transverse electric (TE)- and transverse magnetic (TM)-polarized light extraction efficiencies (LEEs) are sensitive to the spacing and inclined angle for the meshed structure. We also find that the LEE will not be increased when a large filling factor is adopted for the meshed structures, which is because of the competition among the p-GaN layer absorption, the Al metal plasmon resonant absorption, and the scattering effect by meshed structures. The very strong scattering effect occurring in the hybrid p-GaN nanorod/p-AlGaN truncated nanocone contacts can enormously enhance the LEE for both TE- and TM-polarized light, e.g., when the inclined angle is 30°, the LEE for the TE- and TM-polarized light can be increased by ~ 5 times and ~ 24 times at the emission wavelength of 280 nm, respectively.

## Introduction

AlGaN-based deep ultraviolet light-emitting diodes (DUV LEDs) have great application potentials in the scopes such as water purification, medical phototherapy, detection, and photocatalysis [1–3]. However, DUV LEDs with a high external quantum efficiency (EQE) are still difficult to be obtained especially when the emission wavelength decreases. The EQE for LED can be calculated by the product of the internal quantum efficiency (IQE) denoted as *η*_IQE_ and the light extraction efficiency (LEE) denoted as *η*_LEE_, i.e, *η*_EQE_ = *η*_IQE_ · *η*_LEE_. At present, the EQE for conventional flip-chip structured DUV LEDs is lower than 10% which is strongly limited by the low LEE of 7–9% [4]. Thus far, the world-record highest EQE for DUV LEDs is 20% at the wavelength of 275 nm, and such high EQE is achieved thanks to the remarkably enhanced LEE, which is enabled by integrating various advanced LEE technologies such as patterned sapphire substrate, transparent p-electrode, and advanced package technology [5]. Therefore, improving the LEE for realizing high-efficiency DUV LEDs becomes essentially important. It is well known that the LEE is substantially influenced by total internal reflection (TIR) and Fresnel loss, which is caused by the large refractive index contrast between AlGaN and air (*n*_air_ = 1 and *n*_AlGaN_ = 2.6) [6]. In addition, the increase of Al content in AlGaN-based quantum wells yields the dominance of transverse magnetic (TM)-polarized light, which is difficult to propagate into escape cone before being extracted from the DUV LEDs [7]. For increasing the LEE, on one hand, various technologies including roughened surfaces [8], patterned sapphire substrates [9], inclined sidewalls [10], and surface plasma polaritons [11] have been extensively applied, and by doing so, the scattering centers can be generated that helps increase the escape probability from the sapphire substrate for photons. Another obstacle limiting the LEE arises from the absorptive p-GaN contact layer because of the difficulty in growing Al-rich p-AlGaN layer with high hole concentration [5]. Therefore, it is important to reduce the optical absorption that is caused by the p-GaN layer for DUV LEDs, and proposed methods include meshed p-type contact electrode [12, 13], distributed Bragg reflector (DBR)/omni-directional reflector (ODR) [14, 15], and photonic crystal [16]. Among the proposed approaches, meshed p-type contact electrode is effective and less costly. Lobo et al. reported micrometer scale p-type contact patterns and proved to be effective in improving light extraction [13]. However, the investigation of meshed p-type contact electrode of nanometer scale is rarely carried out. Besides that, the scattering effect of the meshed p-type contact electrode of micrometer scale on the LEE is neglected in previous reports. We believe the scattering effect in the nanometer scale p-type contact electrodes can further increase the LEE.

In this paper, the effect of nanoscale meshed contact structure and Al reflector on LEE for DUV LEDs is numerically investigated. Various meshed contact structures are studied including p-GaN nanorod contact, hybrid p-GaN nanorod/p-AlGaN nanorod contacts, and hybrid p-GaN nanorod/p-AlGaN truncated nanocone contacts. By using three-dimensional finite-difference time-domain (3D FDTD) simulation, this work investigates the dependence of LEE on variable parameters for the proposed structures. We find that the LED with optimized hybrid p-GaN nanorod/p-AlGaN truncated nanocone meshed contacts enables over 5-fold and 24-fold LEE enhancement for transverse electric (TE)- and TM- polarized light, respectively.

## Model and Simulation Methods

**Fig. 1:**
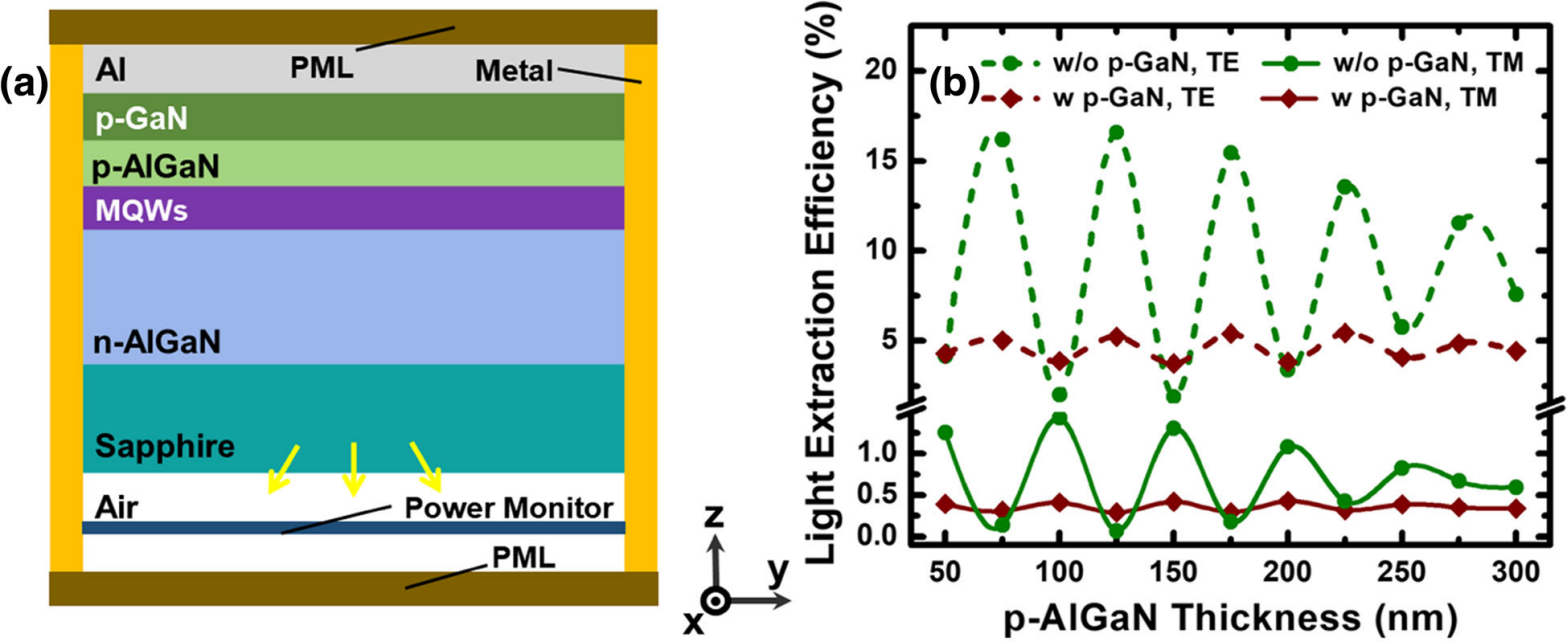
**a** Schematic side view diagram of 3D FDTD computational models for conventional flip-chip DUV LED structure. **b** TE- and TM-polarized LEEs for DUV LED with p-GaN and without p-GaN as a function of the p-AlGaN thickness

The simulator used in our work is developed by Lumerical FDTD solution, which can solve the time-dependent Maxwell’s equations to calculate electromagnetic field distributions in finite structures [17, 18]. Figure 1a presents the simulation model for the conventional flip-chip DUV LEDs. A layer of Al reflector is fixed on the top of the simulated structure for reflecting photons back to the transparent sapphire so that most of the light can be extracted [19]. Note that Al reflector has the reflectivity as high as 92% in the UV spectral range [20]. The metal dissipation mechanism is described by the modified Drude model during the simulation [21]. The thicknesses for the p-GaN layer, the n-AlGaN layer, and the sapphire are set to 100 nm, 1.5 μm, and 1 μm, respectively [12]. Multiple quantum wells (MQWs) are embedded between the n-AlGaN layer and the p-AlGaN layer, for which the total thickness is 100 nm. Besides, we set a single dipole at the middle of the MQWs region and the dipole that represents the electron-hole recombination [22]. The peak emission wavelength of the spectrum for the dipole source is set to 280 nm. The dipole source is polarized in the direction either parallel or perpendicular to the *X*-axis for exciting the TE or TM mode, respectively [23]. The *Z*-axis is perpendicular to the C-plane for DUV LEDs. Therefore, the TE-polarized and the TM-polarized light propagates mainly in the YZ and XY planes, respectively. The absorption coefficients at the emission wavelength of 280 nm for the AlGaN layer, the MQWs, and the GaN layer are assumed to be 10 cm^−1^, 1000 cm^−1^, and 170,000 cm^−1^, respectively. The material refractive indices for the AlGaN layer, the GaN layer, and the sapphire are assumed to be 2.6, 2.9, and 1.8, respectively [23, 24]. The lateral dimension for the calculated structure is set to 8 × 8 μm^2^. The boundary conditions for the four lateral boundaries are assumed to have a reflectance of 100% such that the finite lateral dimensions can be speculated to be infinite [25]. The conditions for the top and the bottom boundaries are set to have perfectly matched layer (PML), which can entirely absorb the electromagnetic energy. In our models, a non-uniform mesh is applied when conducting simulations, and the smallest mesh size is set to 5 nm, which provides good accuracy for calculating the LEE. The power monitor is placed 300 nm apart from the sapphire for collecting the power transmission through the monitor and recording the near-field electric field radiation. The near-field electric field is converted to the far-field electric field by carrying out the Fourier transformation. The LEE is computed by taking the ratio of the total extracted power collected from the power monitor and the total emission power from the dipole [26]. The power collected from the power monitor is obtained by integrating the far-field power distribution over the power monitor surface.

## Results and Discussions

### Effect of the Optical Cavity Thickness on LEE

As it is well known, the optical cavity effect can tune the radiation mode for MQWs in flip-chip LEDs, which is sensitive to the p-type layer thickness, while the p-type layer thickness has a significant influence on LEE [27]. Therefore, we first study the effect of the p-AlGaN layer thickness on the TE-polarized and TM-polarized LEEs for conventional LED structure. The p-AlGaN layer thickness also represents the distance between MQWs and Al reflector. As shown in Fig. 1b, all LEE curves show a periodic oscillation with the p-AlGaN layer thickness and the period is about 50 nm. The oscillating behavior is due to the optical cavity effect that is introduced by the constructive interference between the light from the source and the light reflected by the Al mirror. According to the interference theory, the period can be calculated by Δ*d* = *λ*/2*n*_AlGaN_ = 53 nm [21], which has a good agreement with the simulated results in Fig. 1b. In addition, the peak LEEs for TM-polarized light are opposite to the ones for TE-polarized light. According to Fresnel’s equations and Mueller matrix [28], there are different reflection amplitudes and phase shifts for the reflection of the TE- and TM-polarized light from the interface between two linear isotropic media. Moreover, it can be found that, though the strong p-GaN layer absorption weakens the optical cavity effect, the LEE for LED with 100-nm-thick p-GaN layer still shows a small amplitude fluctuation. The weaker optical cavity effect for LED with p-GaN layer leads to the fact that, for both TE- and TM-polarized light, the minimum LEE for the LED with p-GaN layer is larger than that for the LED without p-GaN layer as shown in Fig. 1b. Meanwhile, it can also be observed that the average LEE for TM-polarized light is only one tenth of that for TE-polarized light, and the findings here are consistent with the results in [23]. Besides, it is worth noting that the LEDs without p-GaN layer show the largest LEEs for TE-polarized light and TM-polarized light to be 16% and 1.5%, respectively, while these numbers are only 5% and 0.5% for the LEDs with p-GaN layer, respectively. Therefore, a threefold enhancement in the LEE can be obtained for the LEDs without a p-GaN layer, which indicates that both TE- and TM-polarized light can be significantly absorbed by the p-GaN layer. It is because some lights need to experience multiple reflections to escape, and the optimized thickness of p-AlGaN also causes the best optical cavity effect. Therefore, reducing the absorption from p-GaN is very important for the LEE of DUV LED and can bring more than double increase in LEE.

### Effect of the Meshed p-GaN Contacts on LEE

**Fig. 2:**
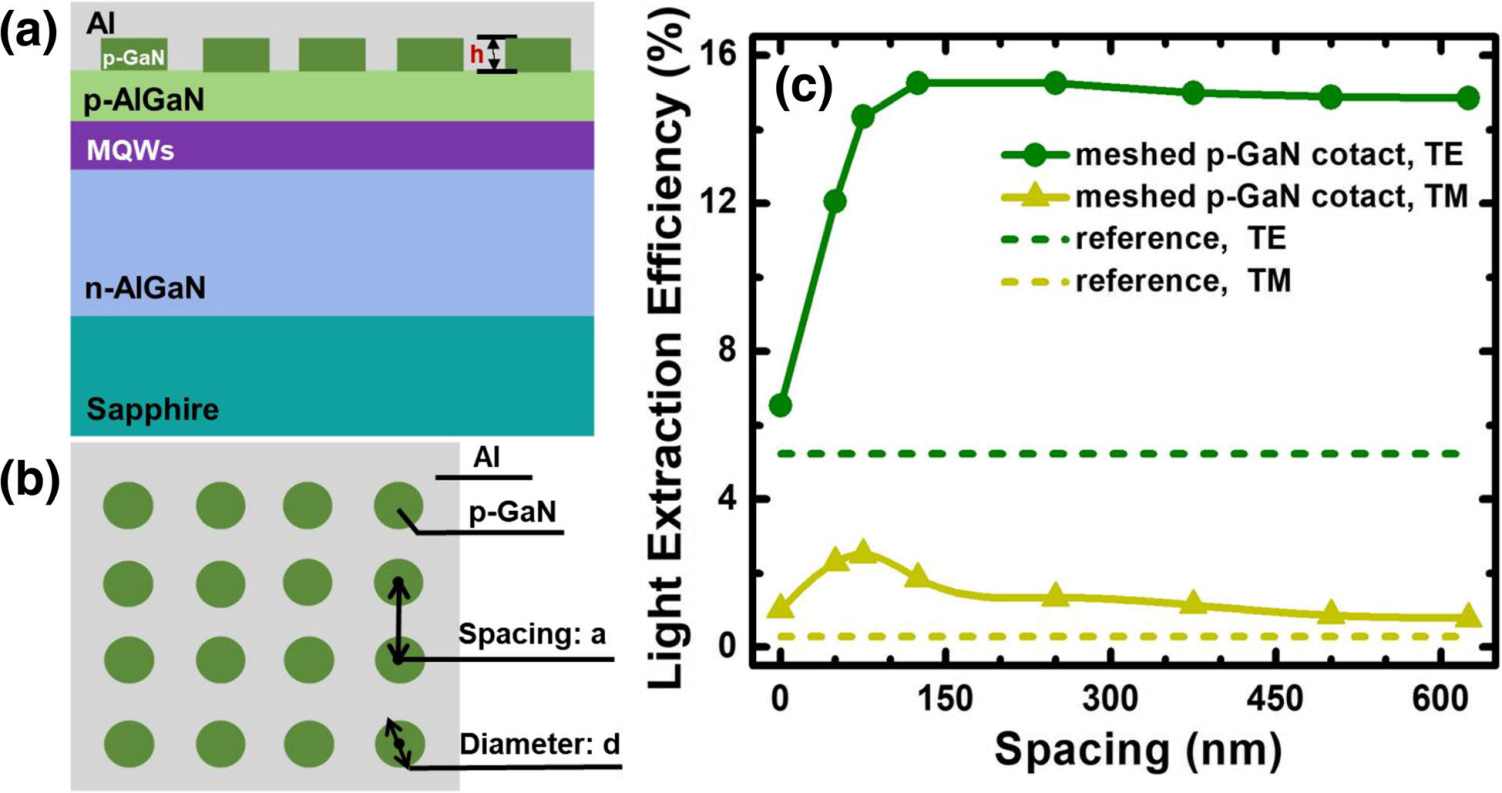
**a** Schematic side view diagram of the flip-chip DUV LED with meshed p-GaN contacts. **b** Schematic top view diagram of meshed p-GaN contact distribution. **c** LEEs for DUV LED with the meshed p-GaN contacts as a function of the spacing of nanorods when the p-AlGaN thickness is 125 nm

To reduce the absorption of the p-GaN layer, p-GaN is meshed into submicro-contacts to increase LEE. Based on the conventional flip-chip DUV LED in Fig. 1a, the p-GaN layer is designed for nanorods which are embedded in the Al reflector to form the p-type submicro-contact electrode (see Fig. 2a) with a square array (see Fig. 2b). The height for p-GaN nanorods is set to 100 nm. The diameter for p-GaN nanorods is fixed at 250 nm which the number is close to the emission wavelength. The optimized p-AlGaN layer thickness is set to 125 nm according to Fig. 1b. For the DUV LED with meshed p-GaN contacts, the spacing is the most important. On the one hand, the smaller spacing shall make the current spread more efficiently into the entire active region. On the other hand, the smaller spacing will increase the filling factor of meshed p-GaN contacts and thus increase the optical absorption. Therefore, an optimized spacing that enables both good current spreading and excellent LEE is very critical for the proposed DUV LEDs. We then investigate and show the effect of spacing on LEE in Fig. 2c. As expected, compared with the conventional DUV LED, the TE-polarized and the TM-polarized LEEs for DUV LEDs with meshed p-GaN contacts are significantly improved. The LEE for the TE-polarized light increases with the increasing spacing until the spacing reaches 125 nm because the absorption of p-GaN decreases as a result of decreasing filling factor of p-GaN. And the LEEs have an over threefold enhancement when the spacing is at around 125 nm. However, after 125 nm, the LEE for the TE-polarized light is decreased with the filling factor. The observations when the spacing is beyond 125 nm infer that there is another factor playing an important effect on LEE. According to the report in [29], the extinction length of the photon can be expressed by 1/*L*_extinction_ = 1/*L*_scattering_ + 1/*L*_absorption_, where *L*_scattering_ and *L*_absorption_ correspond to the scattering length and absorption length, respectively. Because the LEE mainly depends on the material absorption and structural scattering, it can be inferred that the scattering effect caused by the meshed p-GaN contacts dominantly affects the LEE when the spacing is larger than 125 nm.

**Fig. 3:**
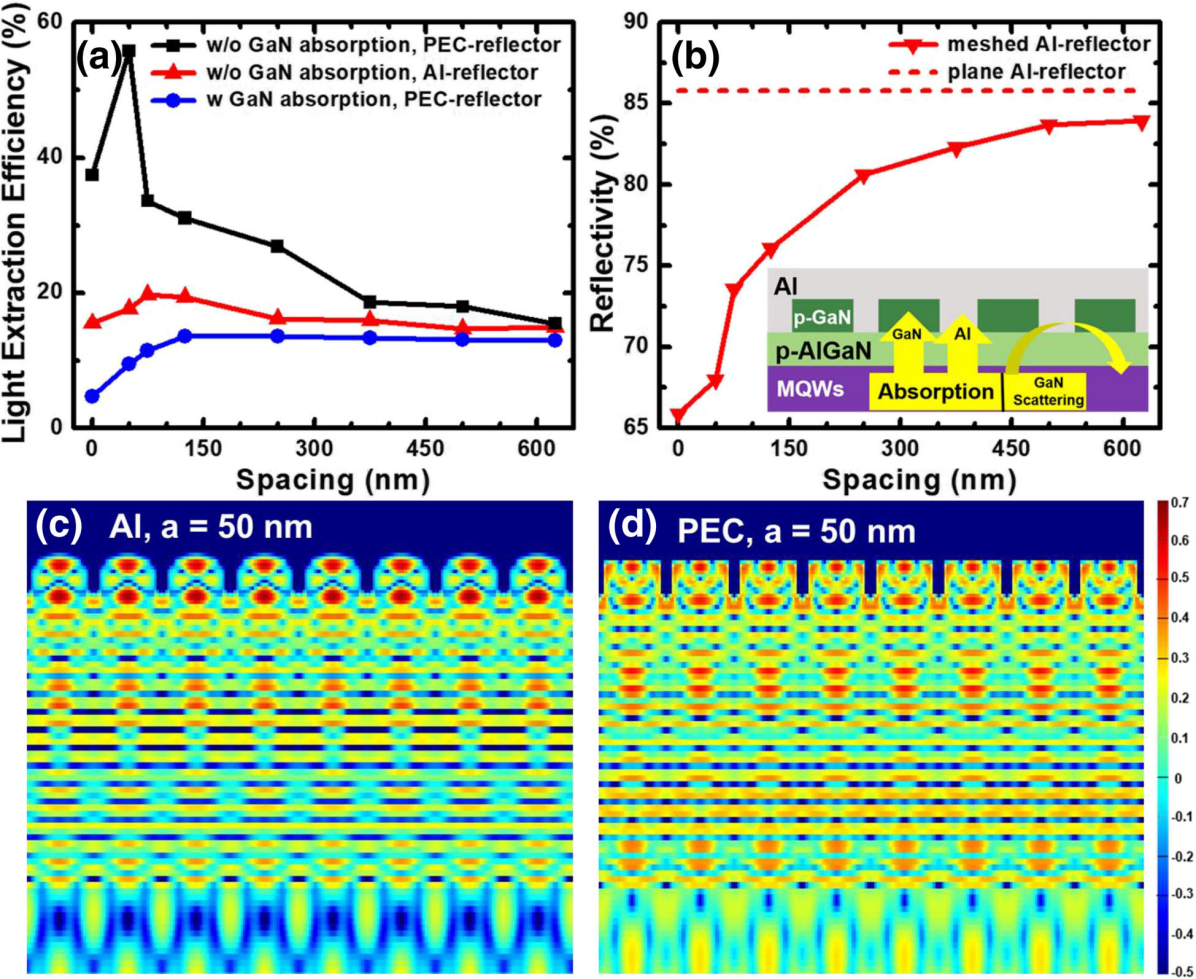
**a** LEEs as a function of the nanorod spacing for DUV LEDs with the 100-nm-high non-absorptive/absorptive p-GaN nanorods, and the reflectors are Al and PEC, respectively. **b** Reflectivity of normal incidence with meshed Al reflector and plane Al reflector as a function of the nanorod spacing. The inset shows the effect of p-GaN layer absorption, Al metal absorption, and structure scattering in DUV LED with meshed p-GaN contacts. Cross-sectional distributions of electric field at the nanorod spacing of 50 nm with **c** Al and **d** PEC reflector

To confirm the scattering effect by the meshed p-GaN contacts, a model without absorptive material is set, such that the absorption coefficient for GaN material is set to 0 and the Al reflector is replaced by the perfect electrical conductor (PEC) with nearly 100% reflectivity, for which the simulation results are plotted with black square line in Fig. 3a. It can be seen that the LEE increases and then decreases with the increased spacing. Namely, the scattering effect of the p-GaN submicro-contact is incremental and then decreases with the increased space. Therefore, the increased spacing for the meshed p-type GaN contacts will suppress the scattering effect, and this interprets the observation in Fig. 3a that when the spacing is larger than 50 nm, the LEE decreases with increased spacing.

In addition, when the absorption of GaN is set to 0 and Al reflector is applied, the LEE also increases firstly and then decreases as the red triangle line shown in Fig. 3a. However, the maximum LEE of 20% for the structure with Al reflector is far smaller than that of 56% for the structure with PEC reflector. Figure 3b presents the dependence of the reflectivity on the spacing for the meshed Al reflector. The reflectivity for meshed Al reflector decreases as the spacing decreases. In another word, the Al metal surface becomes rough when the spacing reduces. Therefore, the decrease in the reflectivity for rough metal surfaces can be attributed to the excitation of surface plasmons and the surface effect [30–32]. Rough metal surface shall modulate the phase of the incident light leading to absorbed light and surface-wave excitation (surface plasmons). Surface effect results in the trapping of the light in the pits of the surface with eventual absorption. Furthermore, the cross-section electric field distribution using plane wave as the incidence source for the Al reflector and PEC reflector is shown in Figs. 3c and d, respectively. It can be found that, for the LED with Al reflector, the p-GaN nanorods possess the strongest local electric field intensity, but such observations are less obvious in the p-GaN nanorods for the LED with PEC reflector, which confirms that there is a surface plasmon resonance absorption for meshed Al reflector. In addition, a similar LEE trend can be observed as the blue circle line in Fig. 3a shows when our model considers the p-GaN layer absorption and PEC reflector. The LEE turns larger for the LED without GaN absorption and with PEC reflector (red triangle line), which indicates that the p-GaN layer absorption is more serious than the metal absorption. Therefore, for DUV LED with meshed p-GaN contacts, there is a competition among p-GaN layer absorption, Al metal absorption, and structure scattering as shown in the insert of Fig. 3b. When the spacing is too small, the LEE is soundly affected by the absorption of the p-GaN layer and the metal, while the structure scattering makes a primary effect on LEE when the spacing becomes large.

**Fig. 4:**
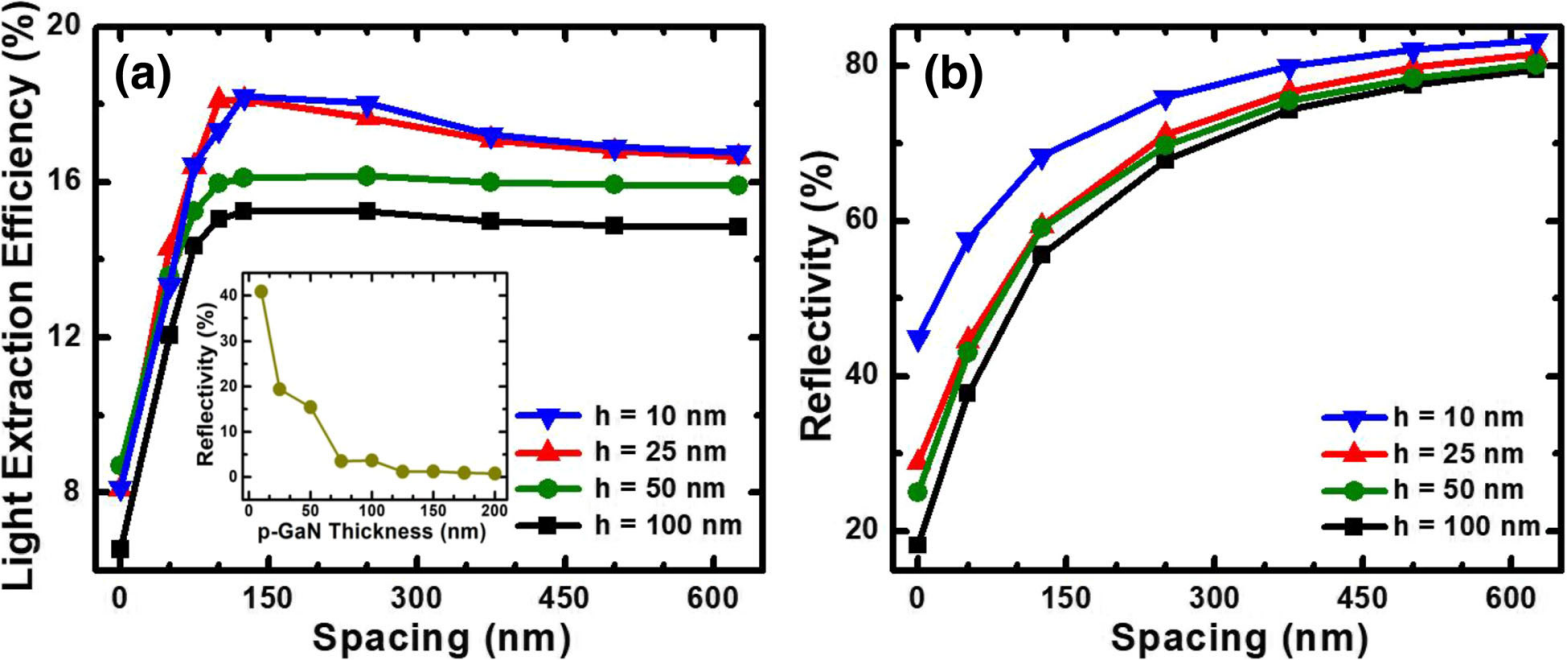
(**a)** LEEs as a function of the nanorod spacing for DUV LED with Al reflector and with the p-GaN nanorod heights of 10, 25, 50, and 100 nm are set. Inset: reflectivity of normal incidence for conventional DUV LED as a function of the p-GaN layer thickness and **b** reflectivity of normal incidence as a function of the nanorod spacing when the p-GaN nanorod heights are of 10, 25, 50, and 100 nm

Besides, we further investigate the effect of p-GaN nanorod height on the LEE for DUV LEDs. Spacing dependence of LEEs at different p-GaN nanorod heights of 10 nm, 25 nm, 50 nm, and 100 nm are shown in Fig. 4a. The LEE increases when the nanorod height decreases from 100 to 25 nm. It is obvious that the increase of LEE is attributed to the weaker absorption of the thinner p-GaN layer. However, Fig. 4a also shows that the LEEs are similar when the nanorod heights are 25 nm and 10 nm. As shown in Fig. 4b, the reflectivity of Al metal with p-GaN nanorods increases faster with the decreased nanorod height. Therefore, it can be inferred that the scattering effect at the 25-nm height is stronger than that at the 10-nm height, which produces similar LEE. Nevertheless, the largest LEE is 15% when the p-GaN nanorods are at the 100-nm height, and the maximum LEE is only 18% when the p-GaN nanorods are at the 25-nm height; thus, a small difference is obtained. It is mainly attributed to the strong absorptive p-GaN layer as shown in the inset of Fig. 4a. For a 10-nm-thick p-GaN layer, only 40% of light can be reflected, so the reflected light is mainly from the Al reflector among the p-GaN nanorods. Consequently, the reflectivity is more affected by spacing rather than the height of p-GaN nanorods. Hence, compared with nanorod spacing, the p-GaN nanorod height less influences the LEE.

### Effect of the Hybrid p-GaN/p-AlGaN Meshed Contacts on LEE

**Fig. 5:**
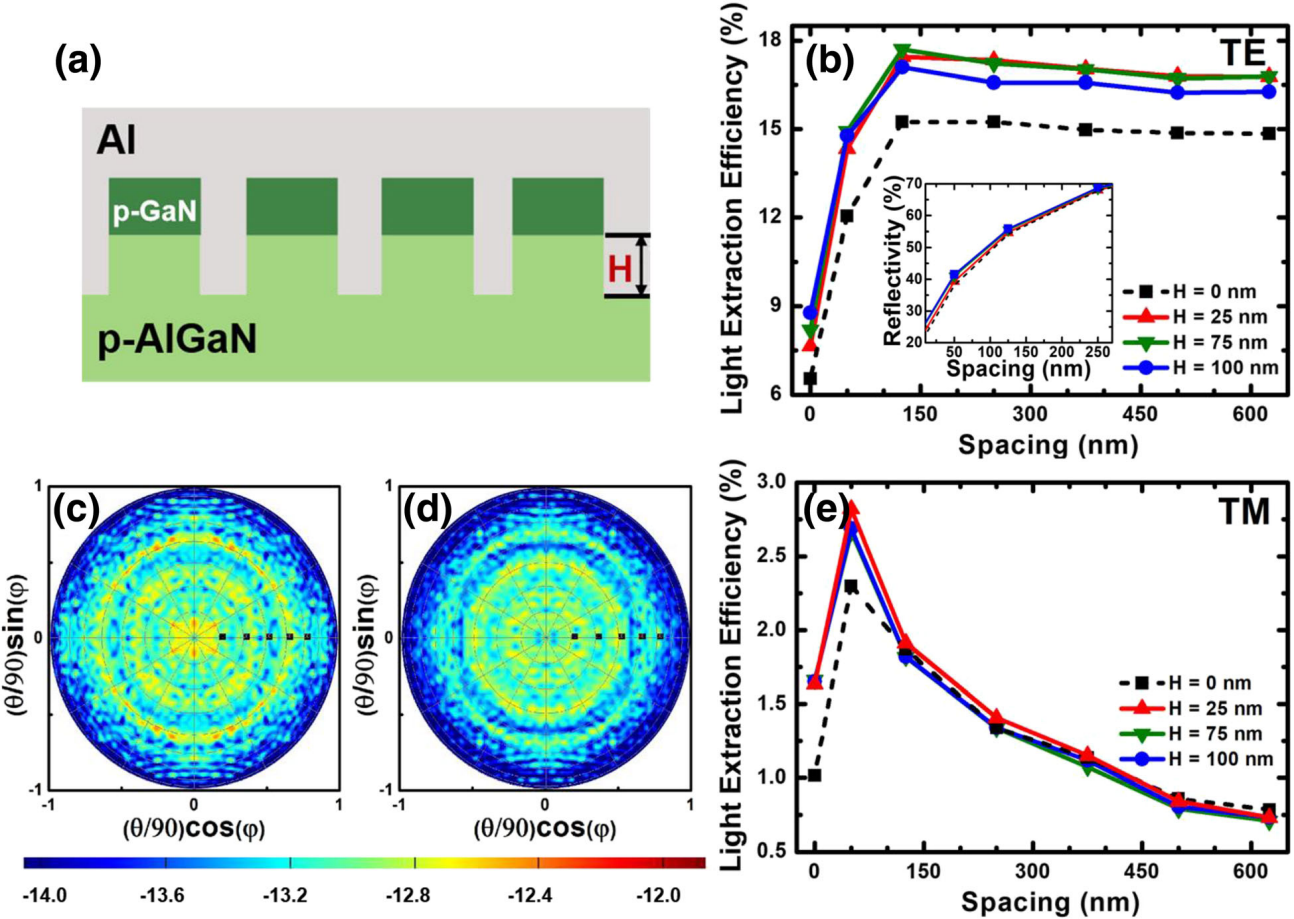
**a** Schematic side view diagram for flip-chip DUV LED with hybrid p-GaN/p-AlGaN nanorod-based meshed contacts. **b** LEEs for TE-polarized light as a function of the nanorod spacing and the p-AlGaN nanorod heights are set to 0, 25, 75, and 100 nm. Inset: Reflectivity of normal incidence for DUV LED with the 100-nm-high p-GaN and with the p-AlGaN height of 0, 25, 75, and 100 nm as a function of the nanorod spacing. Far-field radiation patterns at a spacing of 125 nm with p-AlGaN height of **c** 75 nm and **d** 0 nm. **e** LEEs for TM-polarized light as a function of the nanorod spacing and the p-AlGaN nanorod heights are set to 0, 25, 75, and 100 nm

Furthermore, we further propose hybrid p-GaN/p-AlGaN meshed contacts layer as shown in Fig. 5a. The height and the diameter for p-GaN nanorod are set to 100 nm and 250 nm, respectively. The p-AlGaN nanorod height (*H*) is a variable in this case. The LEEs for different DUV LEDs in terms of nanorod spacing are shown in Fig. 5b, for which we set the values of *H* to 0 nm, 25 nm, 75 nm, and 100 nm. It can be found that the LEEs for DUV LEDs with various high p-AlGaN nanorods are larger than that without p-AlGaN nanorods (*H* = 0 nm). And the LEEs for DUV LEDs are less influenced by the p-AlGaN nanorod height if *H* is not 0 nm. The inset in Fig. 5b shows the normal reflectivity in terms of the nanorod spacing for the hybrid structure, and we can see that the p-AlGaN nanorod height makes a negligible impact on the reflectivity. Therefore, the scattering effect is merely enhanced by p-AlGaN nanorods, which therefore leads to the improved LEE. The far-field radiation patterns for DUV LEDs with 75-nm- and 0-nm-high p-AlGaN nanorods when the nanorod spacing is 125 nm are shown in Figs. 5c and d, respectively. It can be observed that the electric field intensity of DUV LEDs with 75-nm-high p-AlGaN nanorods (see Fig. 5c) is stronger than that with 0-nm-high p-AlGaN nanorods (see Fig. 5d). The electric field distribution for DUV LEDs with 75-nm-high p-AlGaN nanorods is larger than that with 0-nm-high p-AlGaN nanorods, which confirms that the p-AlGaN nanorods improve the scattering effect for light. Figure 5e demonstrates that TM-polarized LEE is even more slightly affected by the p-AlGaN nanorod height.

**Fig. 6:**
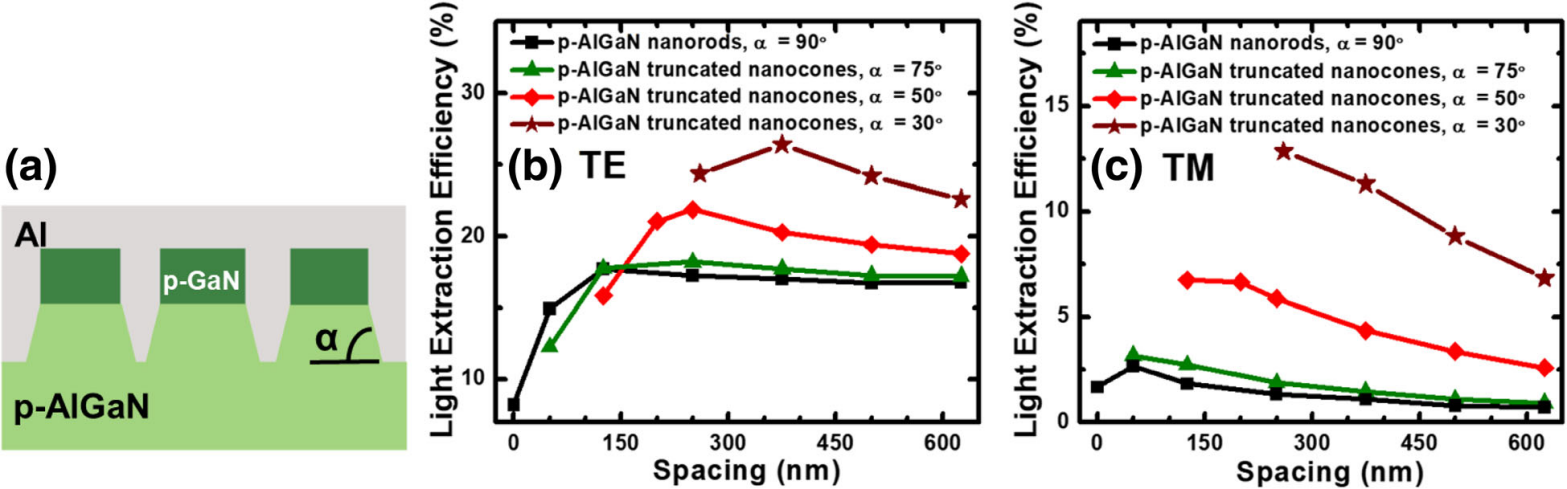
**a** Schematic side view diagram for flip-chip DUV LED with hybrid p-GaN nanorod/p-AlGaN truncated nanocone contacts. LEEs of TE-polarized light (**b**) and of TM-polarized light (**c**) as a function of the nanorod spacing for DUV LEDs, and the structures are with the 100-nm-high p-GaN and 75-nm-high p-AlGaN (the inclined angles are set to 30°, 50°, 75°, and 90°)

Our previous analysis shows that the TM-polarized light is still suffering from extremely low LEE. As a result, methods shall be proposed to scatter the TM-polarized light. For that purpose, we propose p-AlGaN nanorods with inclined sidewalls, thus forming the p-AlGaN truncated nanocones structure as shown in Fig. 6a. The height of the p-AlGaN truncated nanocones is set to 75 nm, and the inclined angle is defined to *α*. A notable LEE enhancement for both TE- and TM-polarized light with the decreased *α* can be seen in Figs. 6b and c, respectively. For the inclined angle *α* = 30°, it is impossible to set a smaller period because the p-AlGaN truncated nanocones have been closely packed when the p-GaN nanorod spacing is 260 nm. The largest TE-polarized LEE reaches 26% when the spacing is 375 nm, and *α* is set to 30°. This number is 1.44 times larger than the design in Fig. 5a. It is more noteworthy that compared with the structure in Fig. 5a, the largest TM-polarized LEE for the design in Fig. 6a is 12% when the spacing is 260 nm and *α* is set to 30°, and this number is increased by 10 times. Compared with the conventional DUV LED without meshed structures, both TE- and TM-polarized LEEs can be increased by over 5 times and 24 times by utilizing the design in Fig. 6a, respectively. These simulated results indicate that p-AlGaN truncated nanocone with an inclined angle of 30° can significantly improve the light scattering effect, especially for TM-polarized light.

## Conclusions

In summary, the impact of various meshed contact structures including p-GaN nanorod, hybrid p-GaN/p-AlGaN nanorod, and p-GaN nanorod/p-AlGaN truncated nanocone on the LEE for DUV LEDs is investigated in detail. It is proven that the p-GaN layer absorption and the Al metal absorption play a main role in the LEE for structures with smaller nanorod spacing, while the scattering ability of meshed structure makes dominating contribution to the LEE for structures with larger nanorod spacing. It is worth noticing that, despite very noticeable LEE enhancement for TE-polarized light, neither the p-GaN nanorod nor the hybrid p-GaN/p-AlGaN nanorod can significantly promote the LEE for the TM-polarized light, which is due to the very poor scattering effect on the in-plane light. Therefore, we further propose and prove that the LEE for the TM-polarized light can be significantly improved by combining p-GaN nanorod and p-AlGaN truncated nanocone, and the optimized inclination angle is found to be 30°. Compared with the conventional DUV LED without meshed structure, a 24-time enhancement in the TM-polarized LEE can thus be achieved.

## Abbreviations

3D FDTD: Three-dimensional finite-difference time-domain method
DBR: Distributed Bragg reflector
DUV LEDs: Deep ultraviolet light-emitting diodes
EQE: External quantum efficiency
IQE: Internal quantum efficiency
LEE: Light extraction efficiency
MQWs: Multiple quantum wells
ODR: Omni-directional reflector
PEC: Perfect electrical conductor
PML: Perfectly matched layer
TE: Transverse electric
TIR: Total internal reflection
TM: Transverse magnetic
